# *Pichia pastoris* protease‐deficient and auxotrophic strains generated by a novel, user‐friendly vector toolbox for gene deletion

**DOI:** 10.1002/yea.3426

**Published:** 2019-07-30

**Authors:** Mudassar Ahmad, Christine M. Winkler, Markus Kolmbauer, Harald Pichler, Helmut Schwab, Anita Emmerstorfer‐Augustin

**Affiliations:** ^1^ Institute of Molecular Biotechnology Graz University of Technology Graz Austria; ^2^ Austrian Centre of Industrial Biotechnology (ACIB) Graz Austria

**Keywords:** auxotrophic strains, gene disruption, knockout plasmids, *P. pastoris*, proteases‐deficient strains

## Abstract

Targeted gene knockouts play an important role in the study of gene function. For the generation of knockouts in the industrially important yeast *Pichia pastoris*, several protocols have been published to date. Nevertheless, creating a targeted knockout in *P. pastoris* still is a time‐consuming process, as the existing protocols are labour intensive and/or prone to accumulate nucleotide mutations. In this study, we introduce a novel, user‐friendly vector‐based system for the generation of targeted knockouts in *P. pastoris*. Upon confirming the successful knockout, respective selection markers can easily be recycled. Excision of the marker is mediated by Flippase (Flp) recombinase and occurs at high frequency (≥95%). We validated our knockout system by deleting 20 (confirmed and putative) protease genes and five genes involved in biosynthetic pathways. For the first time, we describe gene deletions of *PRO3* and *PHA2* in *P. pastoris*, genes involved in proline, and phenylalanine biosynthesis, respectively. Unexpectedly, knockout strains of *PHA2* did not display the anticipated auxotrophy for phenylalanine but rather showed a bradytroph phenotype on minimal medium hinting at an alternative but less efficient pathway for production of phenylalanine exists in *P. pastoris*. Overall, all knockout vectors can easily be adapted to the gene of interest and strain background by efficient exchange of target homology regions and selection markers in single cloning steps. Average knockout efficiencies for all 25 genes were shown to be 40%, which is comparably high.

## 1 INTRODUCTION

Targeted genetic modifications provide the most valuable molecular tools in the study of gene function and have been extensively used to elucidate cellular and molecular processes in yeasts. Traditionally, genes are targeted by linear DNA cassettes that replace the respective locus in vivo by homologous recombination (HR). Sequence information about the target locus is a prerequisite for gene replacement by HR. During the last years, whole genome sequences of important *P. pastoris* strains (e.g., GS115, DSMZ 70382, and CBS 7435) have become available (De Schutter et al., 2009; Mattanovich et al., 2009; Sturmberger et al., 2016), which drastically facilitated genomic engineering of *P. pastoris*. Unfortunately, targeted disruption, insertion, or replacement of genes has proven to be problematic in *P. pastoris* (Schwarzhans et al., 2016). The efficiency of gene replacement was reported to be extremely low, with homologous targeting sequences of <500 bp leading to <0.1% of positive targeting events. Using extended homology regions of >1 kb at each side, this rate could be increased to >50% for selected genes (Näätsaari et al., 2012).

In general, DNA fragments can integrate into the genome via two distinct DNA repair mechanisms that play overlapping roles in yeasts: HR and nonhomologous end joining (NHEJ). HR is mediated through proteins encoded by genes in the Rad52 epistasis group and is generally known to be an accurate repair mechanism, as it involves base‐pairing of long stretches of matched base pairs (Pastwa & Blasiak, 2003). NHEJ requires little to no sequence homology to operate (Krejci, Altmannova, Spirek, & Zhao, 2012). Free DNA ends are first bound by the heterodimer Ku70/80, which in turn recruits the catalytic subunit of DNA protein kinases (DNA‐PKCs; Dudásová et al., 2004; Pastwa & Blasiak, 2003). NHEJ and HR coexist in most cells, but the balance in activity between them varies among species and cell types. For example, accurate HR represents the dominant repair mechanism in the model yeast Saccharomyces cerevisiae, and targeted knockouts can already be achieved with short flanking homology regions of only 40 bp (Brachmann et al., 1998). This property of S. cerevisiae allows construction of knockout cassettes by one‐step polymerase chain reaction (PCR) using 60‐bp primers with 20 bp of their 3′ end binding to the disruptive cassette and 40 bp of its 5′ end being complementary to the target gene (Longtine et al., 1998). Integration events usually occur with >70% efficiency at the correct locus (Gueldener, Heinisch, Koehler, Voss, & Hegemann, 2002). Nonconventional yeasts such as *P. pastoris* often display a high ratio of NHEJ‐to‐HR activity, which is the main reason why targeted gene insertions are difficult to achieve (Näätsaari et al., 2012; Schwarzhans et al., 2016; Tsakraklides, Brevnova, Stephanopoulos, & Shaw, 2015). Several strategies were shown to improve HR activity in *P. pastoris* such as hydroxyurea‐mediated cell cycle arrest (Tsakraklides et al., 2015), increasing the genetic redundancy of host cells by providing extra copies of the gene to be deleted on a helper plasmid (Chen et al., 2013) or deletion of *KU70*, a key player in NHEJ improved targeting (Näätsaari et al., 2012). Deletion of *KU70* was also shown to drastically improved gene modifications using CRISPR/Cas9 tools (Weninger et al., 2018). However, disadvantages such as high complexity, low strain fitness, or not yet fully realized methods for donor cassette integration have kept these techniques from replacing the established ones for the efficient construction of producer strains. Therefore, the most frequently used approach for gene targeting in *P. pastoris* is still the integration of a disruption or expression cassette into the genome via HR. Different strategies have been described for the construction of *P. pastoris* gene targeting cassettes. Homologous flanking regions of ~1 kb are commonly used for the specific targeting of a locus. Combined with a selection marker, this requirement results in cassettes of several thousand base pairs in length, which can be assembled either by cloning (Heiss, Maurer, Hahn, Mattanovich, & Gasser, 2013; Lin‐Cereghino et al., 2006; Nett & Gerngross, 2003; Whittaker & Whittaker, 2005; Wu et al., 2013) or overlap‐extension PCR (oe‐PCR; Näätsaari et al., 2012; Pan et al., 2011). The published cloning methods usually require several subcloning steps and careful selection of appropriate restriction endonucleases. This process can be complicated by low enzyme efficiencies and the fact that required restriction sites might be present multiple times in the PCR product amplified from genomic DNA (gDNA). Additionally, fusion of long DNA fragments by oe‐PCR requires the use of exceptionally long primers for sufficient overlaps, bears the risk of accumulating nucleotide mutations during the amplification process, and often suffers from low yields especially when fusing three or more fragments.

Aside from targeting efficiency, an adequate set of selection markers represents an important factor in targeted gene deletion. Various auxotrophic and antibiotic resistance markers have been described for *P. pastoris* (Cereghino & Cregg, 2000; Lin et al., 2001; Lin‐Cereghino et al., 2008; Nett & Gerngross, 2003; Nett, Hodel, Rausch, & Wildt, 2005; Thor et al., 2005; Whittaker & Whittaker, 2005). Nevertheless, the need for marker recycling is stressed by extensive genetic engineering projects, such as the manipulation of the yeast's glycosylation pathway (Choi et al., 2003; Hamilton et al., 2003). Diverse systems for efficient marker recycling have been established. For example, Nett et al. (2005) and Nett and Gerngross (2003) adapted the Ura‐blaster system for *P. pastoris*, which requires uracil auxotrophy (*ura3*Δ and *ura5*Δ) and resistance to 5‐fluoroorotic acid of the strains. Unfortunately, uracil auxotrophic strains suffer from severe growth retardation, even when grown in media supplemented with uracil (Lin et al., 2001). Other methods for counter selection make use of toxic genes, such as the *T‐urf13* gene from the mitochondrial genome of male‐sterile maize (Soderholm, Bevis, & Glick, 2001) and the Escherichia coli‐derived toxin gene *mazF* (Yang, Jiang, & Yang, 2009). Expression of the toxins exerts strong selection pressure on the transformed cells, stimulating recombination and subsequent loss of the marker cassette. The significant selection pressure, however, causes low cellular viability and might lead to conditional lethality for some gene deletions (Nett & Gerngross, 2003). The stressful effects of toxins can be avoided by employing site‐specific recombinase enzymes for marker recycling. Recombinases trigger the excision of sequences placed between two recombinase target sequences. Näätsaari et al. (2012) placed Flp recombinase under control of the inducible *AOX1* promoter and flanked the marker cassette with 34‐bp Flippase recombination target (FRT) recombination sites. Methanol induction of the *AOX1* promoter resulted in excision of the marker cassette together with the Flp recombinase gene itself. A similar approach using the *Cre‐loxP* system of phage P1 was shown to be likewise applicable in *P. pastoris* (Li et al., 2017; Pan et al., 2011).

In this study, we introduce a simple and potent system to create knockout cassettes for gene targeting in *P. pastoris*. PCR‐amplified homology sequences are integrated into a vector in a single cloning step. Highly efficient cloning is enabled by the specific properties of the employed *Sfi*I restriction endonuclease. The method introduced here allows effortless exchange of selection markers within the targeting vector, while obviating the need for amplification of long DNA fragments by PCR, a notoriously laborious, comparably expensive and error‐prone process. In combination with the Flp recombinase system for marker recycling described above, our system is applicable for repeated and consecutive gene deletions. We demonstrate the efficiency of our approach by reproducing already described gene deletions of, for example, *LYS2* (Austin, Kuestner, Chang, Madden, & Martin, 2011), *MET2* (Thor et al., 2005), *TYR1* (Whittaker & Whittaker, 2005), *SUB2* (Salamin, Sriranganadane, Léchenne, Jousson, & Monod, 2010), *PEP4* and *PRB1* (Gleeson, White, Meininger, & Komives, 1998), *PRC1* (Ohi, Ohtani, Okazaki, Furuhata, & Ohmura, 1996), *YPS1*, *YPS2*, and *YPS7* (Guan et al., 2012), *KEX1* (Boehm, Pirie‐Shepherd, Trinh, Shiloach, & Folkman, 1999), and *KEX2* (Werten & De Wolf, 2005). Additionally, we targeted so‐far uncharacterized, putative genes we selected from sequence annotations (Küberl et al., 2011) and blast searches (https://blast.ncbi.nlm.nih.gov) specifically looking for potential protease activity and/or the presence of a secretion signal sequence (e.g., *PrtP*, *CTSE*, and *KPX1‐KPX26* for Knockout Protease X). The rationale behind this was to create a set of protease‐deficient strains to be tested for improved recombinant expression of proteins in future experiments, because endogenous protease activity was reported to cause proteolysis of heterologous proteins (Ahmad, Hirz, Pichler, & Schwab, 2014; Macauley‐Patrick, Fazenda, McNeil, & Harvey, 2005). In order to generate new auxotrophic strains, we deleted the biosynthetic genes *PRO3* and *PHA2*. Deletion of *PRO3* resulted in a *P. pastoris* strain auxotrophic for proline. Knocking‐out *PHA2* did not lead to complete phenylalanine auxotrophy as previously reported for S. cerevisiae (Braus, 1991), which suggests that an alternative route must exist for the biosynthesis of phenylalanine in *P. pastoris*. Our deletion vectors pPpKC1–4 and all protease‐deficient and auxotrophic strains described in this study have been deposited at and are available from the *Pichia Pool* (Ahmad et al., 2014) at the Institute of Molecular Biotechnology, Graz University of Technology.^1^

## 2 MATERIALS AND METHODS

### 2.1 Strains and media

E. coli Top 10F′ (Life Technologies, Carlsbad, CA) was used for all cloning steps. *P. pastoris* CBS 7435 wild type (NRRL‐Y11430 and ATCC 76273), CBS 7435 *his4*, and CBS 7435 *arg4* (Näätsaari et al., 2012) strains were used as hosts for genetic modifications. Phusion polymerase, DNA modifying enzymes, DNA ladder, and plasmid DNA isolation kit were purchased from Thermo Scientific (Bremen, Germany). T4 DNA Ligase and Wizard^®^ SV Gel PCR Clean‐Up System were obtained from Promega (Madison, WI). l‐Lysine–HCl, l‐phenylalanine, and l‐proline were purchased from SERVA Electrophoresis (Heidelberg, Germany). l‐Arginine–HCl, l‐histidine, l‐methionine, and l‐tyrosine were purchased from Carl ROTH GmbH (Karlsruhe, Germany). Zeocin^™^ was purchased from InvivoGen (Eubio, Vienna, Austria). All other chemical reagents used in this study were purchased from Lactan (Graz, Austria). E. coli media components were obtained from AppliChem (VWR International GmbH, Vienna, Austria). E. coli was cultivated on Luria–Bertani medium (1% tryptone, 0.5% yeast extract, 0.5% NaCl, and 2% agar) supplemented with 100 μg/ml of ampicillin or 25 μg/ml Zeocin^™^. *P. pastoris* media components were from BD Biosciences (Becton Dickinson GmbH, Vienna, Austria). *P. pastoris* was grown in buffered yeast extract peptone dextrose medium (BYPD; 2% peptone, 1% yeast extract, 2% glucose, 200‐mM potassium phosphate, and pH 7.0) or buffered minimal dextrose medium (BMD; 1.34% yeast nitrogen base without amino acids; 4 × 10^−5^% biotin, 2% dextrose, 200‐mM potassium phosphate, and pH 7.0, supplemented with or without respective amino acids). Auxotrophic knockouts *tyr1*Δ, *pro3*Δ, and *pha2*Δ were grown on BMD media without or with respective amino acids as these knockout strains were, except for the latter, not able to grow in rich media (Brandriss, 1979; Whittaker & Whittaker, 2005). For marker recycling, transformants were cultivated in buffered minimal methanol (BMM; 1.34% yeast nitrogen base without amino acids; 4 × 10^−5^% biotin, 0.5% methanol, 200‐mM potassium phosphate, and pH 7.0, with or without amino acid supplementation).

### 2.2 Construction of knockout vector backbone

**Figure 1:**
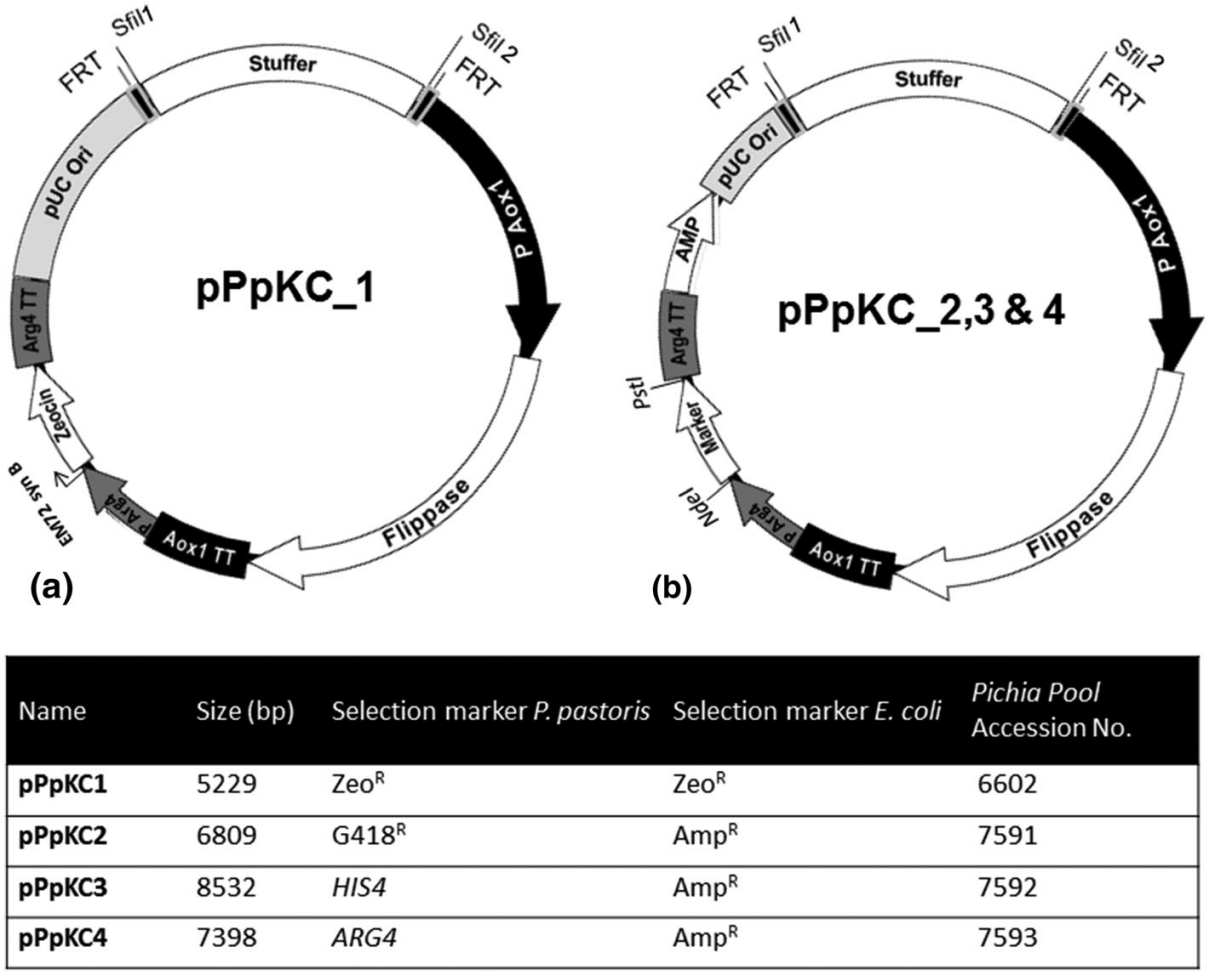
Knockout plasmids harbouring different Pichia pastoris selection markers constructed and applied during this study. (a) pPpKC1. (b) pPpKC2–4. Indicated are the unique restriction sites NdeI and PstI for marker exchange

Sequences of all primers used in this study are given in Table S1. The four basic knockout plasmids (pPpKC1–4) harbouring different selection markers were constructed during this study (Figure 1A,B). The plasmid pPpT4 (JQ519689; Näätsaari et al., 2012) was used as initial backbone to construct the pPpKC1 knockout plasmid. The origin and function of different components used to construct these basic knockout plasmids are given in Table S2. A synthetic DNA fragment, denoted as “stuffer,” was amplified by PCR from plasmid pAaHBglHRP0 with primers *Pci*IFRT*Sfi*I1F and *Bgl*IIFRT*Sfi*I2R (HPLC purified), digested with *Pci*I and *Bgl*II and cloned into the pPpT4 vector. Different components of the knockout plasmid pPpKC1 were amplified and joined by oe‐PCR followed by classical restriction enzyme cloning using strategically placed restriction sites (*Pci*I, *Bgl*II, and *Nco*I). Equimolar ratio of different PCR products was used for oe‐PCR. The vector backbone pPpKC1 was completely sequenced (Microsynth AG, Vienna, Austria). Plasmids derived from pPpKC1 were sequenced for exchanged parts. The marker cassette *KanMX* (consisting of argininosuccinate lyase [*ARG4*] promoter, synthetic *KanMX* coding sequence, and *ARG4* terminator) was amplified from pAKBgl expression plasmids (Ahmad et al., 2014), and *Pvu*II*‐Avr*II cloned into pPpKC1 to generate pPpKC2. The *HIS4* (phosphoribosyl‐ATP pyrophosphatase, phosphoribosyl‐AMP cyclohydrolase, and histidinol dehydrogenase, X56180) and *ARG4* coding sequences were obtained by restricting pAHBgl and pAABgl expression plasmids with *Nde*I*‐Pst*I and were cloned into pPpKC2 using the same restriction enzymes to generate pPpKC3 and pPpKC4, respectively. Sequences are provided in File S3 (.gb formate).

### 2.3 Construction of knockout cassettes

**Figure 2:**
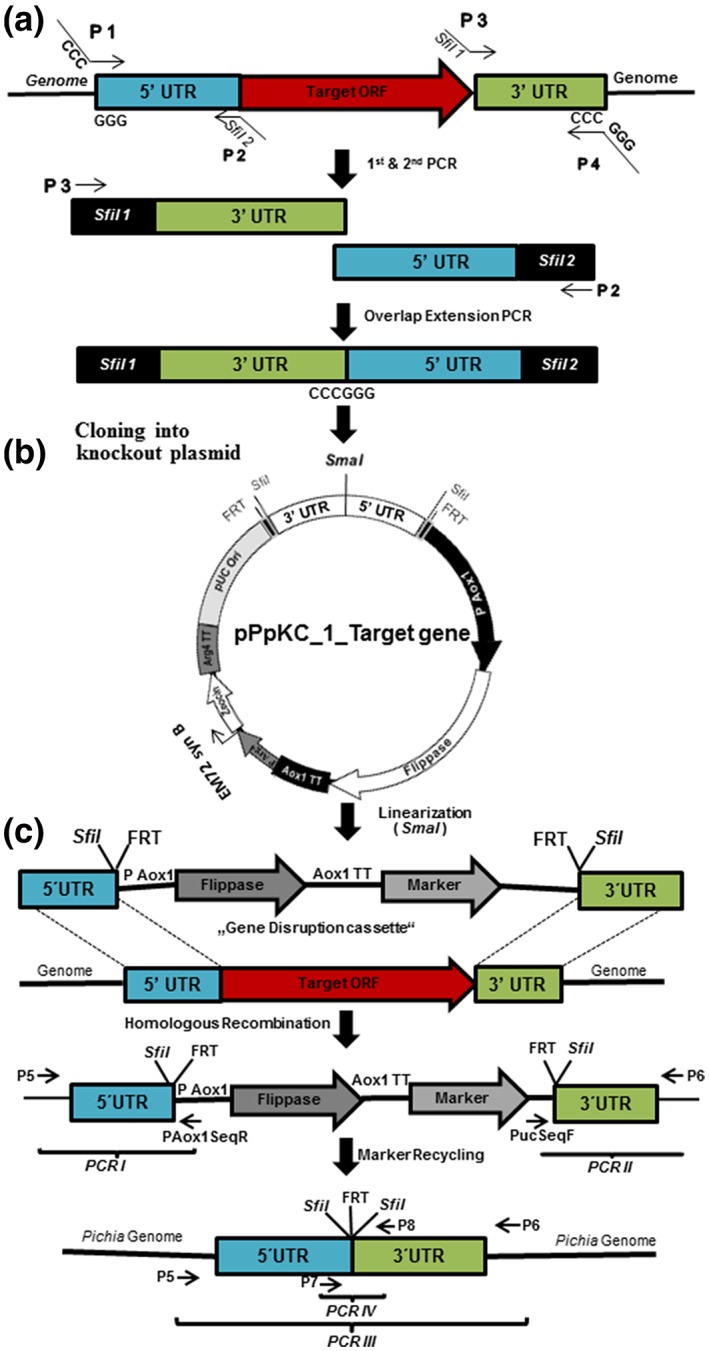
Schematic representation of the experimental procedure for gene deletion and its confirmation. (a) By performing two PCR reactions, the 3′‐ and 5′‐homology regions of the respective target gene were amplified separately. The two PCR products were joined by oe‐PCR, creating a unique restriction site (SmaI) for subsequent linearization. (b) The SfiI‐restricted 3′‐ and 5′‐homology regions were cloned into the knockout vector. The final knockout vector was linearized using SmaI prior to transformation into Pichia pastoris. (c) Homologous recombination replaced the target open reading frame (ORF) with the linear knockout cassette. The correct integration was verified by amplifying region I containing the 5′‐homology (primer pair P5/PAox1SeqR) and region II containing the 3′‐homology (primer pair PucSeqF/P6). Clones with positive results for both PCRs were selected for marker recycling. The removal of the integrated marker cassette was verified by amplification of regions III and IV. FRT, flippase recombination target site; P1–P8, primers

`To construct the knockout cassettes, 5′‐ and 3′‐homology regions were amplified from gDNA of *P. pastoris* CBS 7435 in two separate PCR reactions and merged in one oe‐PCR (Figure 2a). The exact lengths of the amplified homology regions are given in Table 1. Two sets of primers (P1/P2 for 5′‐homology and P3/P4 for 3′‐homology) were used for amplification of homology regions for each target gene. Apart from a sequence complementary to the target locus, the primers were designed to have the following features: the primers P2 and P3 contained the “*Sfi*I 2” (5′‐GGCCGATCAGGCC‐3′) and “*Sfi*I 1” (5′‐GGCCACTAGGGCC‐3′) recognition sequences, respectively. The forward primer (P1) for 5′‐homology and reverse primer (P4) for 3′‐homology contained sequences complementary to each other (~20 nucleotides) for oe‐PCR. Their binding sites on the genome sequence were selected in a way that merging of the two fragments generates a unique blunt end restriction enzyme site, for example, *Sma*I, for subsequent linearization of the knockout cassette (Figure 2a). In principle, any blunt end restriction enzyme site, which is not present in the backbone, can be used for this purpose. The fragment resulting from oe‐PCR was digested with *Sfi*I and ligated into the knockout vector backbone (Figure 2b). Ligation was confirmed by colony PCR using primers PucSeqF and PAox1SeqR, in addition to restriction analysis with *Sfi*I. Correct cloning of the insert was also confirmed by sequencing. Sequences are provided in file S3.

**Table 1:** Knockout efficiencies for biosynthetic and protease genes

#	Deleted gene	Protein ID CBS 7435	Protein size (a.a.)	Signal peptide	Selection marker	% targeting efficiency	Closest Saccharomyces cerevisiae homologue	Blastp e‐value^d^
Biosynthetic gene knockouts—phenotypic growth analysis on selective media								
1	*met2*Δ^a^	CCA40261.1	475	—	Zeocin^™^	14.9	Met2p	1.4e‐134
2	*lys2*Δ^a^	CCA37057.1	1,400	—	Zeocin^™^	4.2	Lys2p	0
3	*pro3*Δ	CCA40748.1	274	—	Zeocin^™^	33.7	Pro3p	1.1e‐70
4	*tyr1*Δ	CCA38031.1	431	—	Zeocin^™^	8.5	Tyr1p	1.1e‐141
5	*pha*Δ*2* b	CCA40709.1	299	—	Zeocin^™^	47.2	Pha2p	1.8e‐51
Protease gene knockouts—confirmation by PCR								
1	*sub2*Δ	CCA37470.1	477	Yes	Zeocin^™^	20.0	Prb1p	4.2e‐96
2	*pep4*Δ^c^	CCA39046.1	410	Yes	*HIS4*	72.0	Pep4p	1.5e‐157
3	*prb1*Δ	CCA36690.1	559	Yes	Zeocin^™^	68.0	Prb1p	7.3e‐147
4	*prc1*Δ	CCA36928.1	523	Yes	Zeocin^™^	8.0	Prc1p	1.1e‐173
5	*yps1*Δ	CCA40555.1	599	No	Zeocin^™^	88.0	Yps1p	1.3e‐95
6	*yps2*Δ	CCA39867.1	527	Yes	Zeocin^™^	33.6	Yps1p	5.8e‐44
7	*yps7*Δ	CCA39772.1	582	Yes	Zeocin^™^	36.7	Yps7p	1.8e‐23
8	*kex1*Δ	CCA38812.1	624	Yes	Zeocin^™^	36.7	Kex1p	2.8e‐90
9	*kex2*Δ^c^	CCA38676.1	777	Yes	*HIS4*	56.3	Kex2p	6.8e‐176
10	*prtP*Δ	CCA38447.1	1,810	Yes	Zeocin^™^	26.7	Flo10p	8.2e‐08
11	*ctse*Δ	CCA36842.1	536	No	Zeocin^™^	32.0	Mkc7p	5e‐41
12	*kpx1*Δ	CCA40794.1	327	Yes	Zeocin^™^	8.0	Ent2p	0.99
13	*kpx4*Δ	CCA39283.1	612	Yes	Zeocin^™^	6.0	Mkc7p	1.9e‐30
14	*kpx8*Δ	CCA40011.1	593	Yes	Zeocin^™^	48.0	Yps1p	6.9e‐48
15	*kpx10*Δ	CCA38814.1	1,610	Yes	Zeocin^™^	72.0	Flo10p	8.9e‐08
16	*kpx17*Δ	CCA39747.1	578	Yes	Zeocin^™^	76.0	Ecm14p	5.7e‐108
17	*kpx20*Δ	CCA40153.1	587	Yes	Zeocin^™^	56.7	Cwp1p	0.034
18	*kpx21*Δ	CCA40152.1	1,474	Yes	Zeocin^™^	42.7	Flo10p	8.5e‐10
19	*kpx24*Δ	CCA36885.1	276	No	Zeocin^™^	26.7	Srt1p	1.4e‐47
20	*kpx25*Δ	CCA39190.1	990	No	Zeocin^™^	63.3	Pff1p	1.9e‐145

a The targeting efficiencies for *MET2* and *LYS2* loci were averaged from transformations into different strain backgrounds, for example, wild type, *his4*Δ, and *arg4*Δ.

b Eighty‐three of a total 176 transformants showed leaky and retarded growth on BMD media. Twenty‐four of these 83 growth‐retarded transformants were screened for the integration of the knockout cassette into the correct locus, and all of them were positive. Therefore, we assumed that all 83 clones with retarded growth were successful *pha2*Δ knockouts.

c Knockout was not successful with Zeocin^™^ marker of pPpKC_1.

d e‐values represent homology to the closest *Saccharomyces cerevisiae* homologue performed at *Saccharomyces* Genome Database (SGD; http://www.yeastgenome.org/).

### 2.4 Transformations

*P. pastoris* competent cells were prepared using the condensed protocol (Lin‐Cereghino et al., 2005). For most efficient HR, 1–2 μg of linear DNA cassettes were transformed into competent cells using electroporation. Immediately after electroporation, 500 μl of 1‐M sorbitol and 500 μl of YPD or BMD‐AA (*pro3*Δ, *tyr1*Δ, and *pha2*Δ knockouts) were added, and cells were allowed to regenerate for 2 hr at 28°C and 120 rpm. Transformants carrying the Zeocin^™^ marker were selected on YPD plates supplemented with 25 μg/ml Zeocin^™^ or BMD‐AA plates supplemented with 100 μg/ml Zeocin^™^. For selection of *KanMX* marker transformants, the concentration of G418 in the media was 300 mg/L. Amino acids were generally supplemented to a concentration of 150 mg/L, except for histidine, which was added to 40 mg/L.

### 2.5 Characterization of knockout strains

For analysis of gene knockouts resulting in auxotrophies (*pha2*Δ, *met2*Δ, *lys2*Δ, *pro3*Δ, and *tyr1*Δ), single colonies of transformants were picked to inoculate 250 μl of BMD‐AA in 96‐well deep well plates (DWPs) and were grown for 24 hr at 28°C and 320 rpm. The cultures were pinned onto BMD, BMD‐AA, and YPD plates to calculate the targeting efficiencies for each locus based on fast/slow growth (*pha2*Δ) or growth/no growth phenotypes (*met2*Δ, *lys2*Δ, *pro3*Δ, and *tyr1*Δ). Protease gene knockouts lacking an easily identifiable phenotype were confirmed by PCR only. A fast protocol for isolation of gDNA was applied (Lõoke, Kristjuhan, & Kristjuhan, 2011). For confirmation of correct, site‐specific integration, two independent PCR reactions, namely, PCR I and PCR II, were performed. As shown in Figure 2c, the outer primers P5 and P6 bind ~100 bp outside of the 5′‐ and 3′‐homology regions selected for HR, whereas the inner primers PAox1SeqR and PucSeqF bind the *AOX1* promoter and *pUC* origin of replication, respectively. A PCR product is obtained only if integration has occurred at the right locus. In a first step, transformants were screened for the 5′‐homology region (PCR I). Clones, which showed correct integration, were examined in a second PCR by using primers for the 3′‐homology region (PCR II). Transformants showing correct integration on both sides of the target locus were retrieved from the backup library; gDNA of the respective strain was isolated and reconfirmed by PCR reactions I, II, III, and IV (Figure 2c).

### 2.6 Marker recycling

To start expression of Flp recombinase from P_*AOX1*_, and thereby recycling of the selection marker, transformants were cultivated in 50 ml of BMM media at 28°C and 120 rpm. After 24 and 48 hr of induction, cultures were streaked onto nonselective media to generate single colonies. Cells arising from single colonies were cultivated in 96‐well DWPs and were screened for the removal of the marker by pinning onto selective and nonselective agar medium, respectively. The marker recycling efficiencies were calculated as percentage of the colonies that had lost the marker cassettes.

### 2.7 Growth rate studies

The growth rate of *P. pastoris* wild type and knockout strains *met2*Δ, *lys2*Δ, *pro3*Δ, *tyr1*Δ, and *pha2*Δ was analysed by measuring the optical density (OD_600_) in triplicate of cultures grown in 50 ml of BYPD or BMD media with our without supplementation of respective amino acids in 300‐ml baffled flasks.

## 3 RESULTS

### 3.1 Construction of knockout vector backbones

The strategy to recycle selection markers based on the Flp/FRT recombinase system was first described by Wirsching, Michel, and Morschhäuser (2000) and later optimized by Näätsaari et al. (2012) for use in *P. pastoris*. In both protocols, the knockout cassette was assembled and amplified by oe‐PCR, a process prone to mutations. In the present study, we aimed at constructing knockout vectors that can be linearized at a unique restriction site to give the final knockout cassette containing the Flp/FRT marker recycling system. To achieve this goal, the Flp recombinase expression cassette, Zeocin^™^ resistance cassette and E. coli origin of replication were flanked by two 34‐bp FRT repeats. We cloned a stuffer fragment, flanked by two *Sfi*I restriction sites (GGCCNNNN/NGGCC), in between of these FRT repeats to construct the knockout plasmid pPpKC1 (Figure 1a). The single‐stranded overhangs generated by the *Sfi*I restriction enzyme were designed to be incompatible to each other to prevent religation of restricted backbone and to facilitate directional cloning of the insert. We hence termed these sites *Sfi*I 1 and *Sfi*I 2. The special feature of *Sfi*I restriction endonuclease, a type IIF restriction enzyme, is that it interacts with two restriction sites simultaneously and cleaves them in a concerted manner, guaranteeing high restriction efficiencies (Wentzell, Nobbs, & Halford, 1995). Indeed, we observed exceptionally high ligation efficiencies of more than 95% with *Sfi*I‐cut vectors and inserts (data not shown). Furthermore, we exchanged the Zeocin^™^ marker gene of pPpKC1 with alternative *P. pastoris* markers *KanMX*, *HIS4*, and *ARG4* to expand the versatility of the system. The latter three yeast markers were combined with an ampicillin resistance marker for selection in E. coli. These modifications yielded the knockout vectors pPpKC2, pPpKC3, and pPpKC4, respectively (Figure 1b). We included the same *Sfi*I 1 and *Sfi*I 2 recognition sequences in all the constructed knockout vectors, thereby promoting effortless exchange of target homology regions between them.

The marker cassette of each plasmid was tested for functionality by transforming adequate *P. pastoris* strains and selecting transformants on respective media. As routinely applied for most of our *Pichia Pool* plasmids (Ahmad et al., 2014), *ARG4* promoter/terminator sequences were used to drive expression of all marker genes in these knockout plasmids. *ARG4* promoter/terminator sequences are extremely short, and the promoter strength is comparably weak, which allows lower amounts of antibiotics to be used when screening for correct cassette integration and helps keep undesired multicopy insertion events to a minimum. Also, when using *ARG4* promoter/terminator sequences, we never observed reduced transformation efficiencies or malformed colonies as reported when using the pPpT4 plasmid, which harbours a strong *ILV5* promoter to drive marker gene expression (Näätsaari et al., 2012). The presence of homologous sequences, that is, *ARG4* promoter/terminator did not result in reduced integration efficiencies when targeting the *AOX1* locus. On the contrary, we observed high HR frequencies when targeting the *AOX1* locus using this marker cassette in routine protein expression experiments (own unpublished observations).

### 3.2 Tailoring of knockout vectors

The applicability and effectiveness of our knockout vector approach were verified by targeting five biosynthetic genes (*MET2*, *LYS2*, *PRO3*, *TYR1*, and *PHA2*) and 20 known or putative protease genes (Table 1). To target the knockout cassettes to these loci, we amplified approximately 1,000 bp of the respective 5′‐ and 3′‐regions from gDNA of wild type *P. pastoris* CBS 7435. During this PCR step, the restriction sites *Sfi*I 1 and *Sfi*I 2 were added on primers. These restriction sites were later used for cloning of the target homology regions into the knockout vector backbones. The two amplified 5′‐ and 3′‐homology fragments were joined by oe‐PCR, thereby introducing a unique blunt end restriction site, for example, *Sma*I, between the fragments that could later be used for linearization of the vector. We generated this unique *Sma*I restriction site by choosing the binding position of the outermost primers on the genome sequence, P1 and P4, in a way that they reconstitute the recognition sequence for the blunt end restriction enzyme after fusion in the oe‐PCR (Figure 2a). Following restriction with *Sfi*I, the product of oe‐PCR was cloned into the vector backbone pPpKC1 (Figure 2b).

### 3.3 Construction and characterization of auxotrophic knockout strains

Knockout plasmids based on pPpKC1 and harbouring 5′‐ and 3′‐homology regions to target *MET2*, *LYS2*, *PRO3*, *TYR1*, and *PHA2* were linearized at the unique *Sma*I site. The resulting linear knockout cassettes were applied to transform CBS 7435 wild type cells to create strains auxotrophic for a single amino acid. Alternatively, the knockout cassettes were used to transform CBS 7435 *his4*Δ or *arg4*Δ (Näätsaari et al., 2012) to create double auxotrophic strains. In summary, we created nine single or double auxotrophic strains, namely, *met2*Δ, *met2*Δ *arg4*Δ, *met2*Δ *his4*Δ, *lys2*Δ, *lys2*Δ *arg4*Δ, *lys2*Δ *his4*Δ, *pro3*Δ, *tyr1*Δ, and *pha2*Δ. An advantage of targeting these genes is the simple and reliable confirmation of the knockout based on the growth phenotype on minimal medium. Transformants of pPpKC1_*MET2*‐ and *LYS2*‐ knockout cassettes were selected on YPD + Zeocin^™^. Whittaker and Whittaker (2005) reported the inability of *P. pastoris tyr1*Δ to grow on rich complex media, that is, YPD. The same phenotype was observed for S. cerevisiae *pro3*Δ by Brandriss (1979). Accordingly, we selected for *tyr1*Δ, *pro3*Δ, and *pha2*Δ transformants on BMD + Zeocin^™^. The efficiency of gene targeting was assessed by pinning the transformants on selective and nonselective media in parallel. A transformant was classified to carry a successful knockout if it showed growth on BMD supplemented with the respective amino acid (BMD‐AA) but not on BMD alone. The calculated average gene targeting efficiency was shown to be 40%, whereas efficiencies for deletion of single genes ranged from 4% to 88% (Table 1). As the length of the homology regions was fairly similar for all targeted genes, the strong variation in targeting efficiency between the different loci must result from other factors, such as stability of disruption cassettes, DNA superstructures of gene loci, or off‐target recombination events.

**Figure 3:**
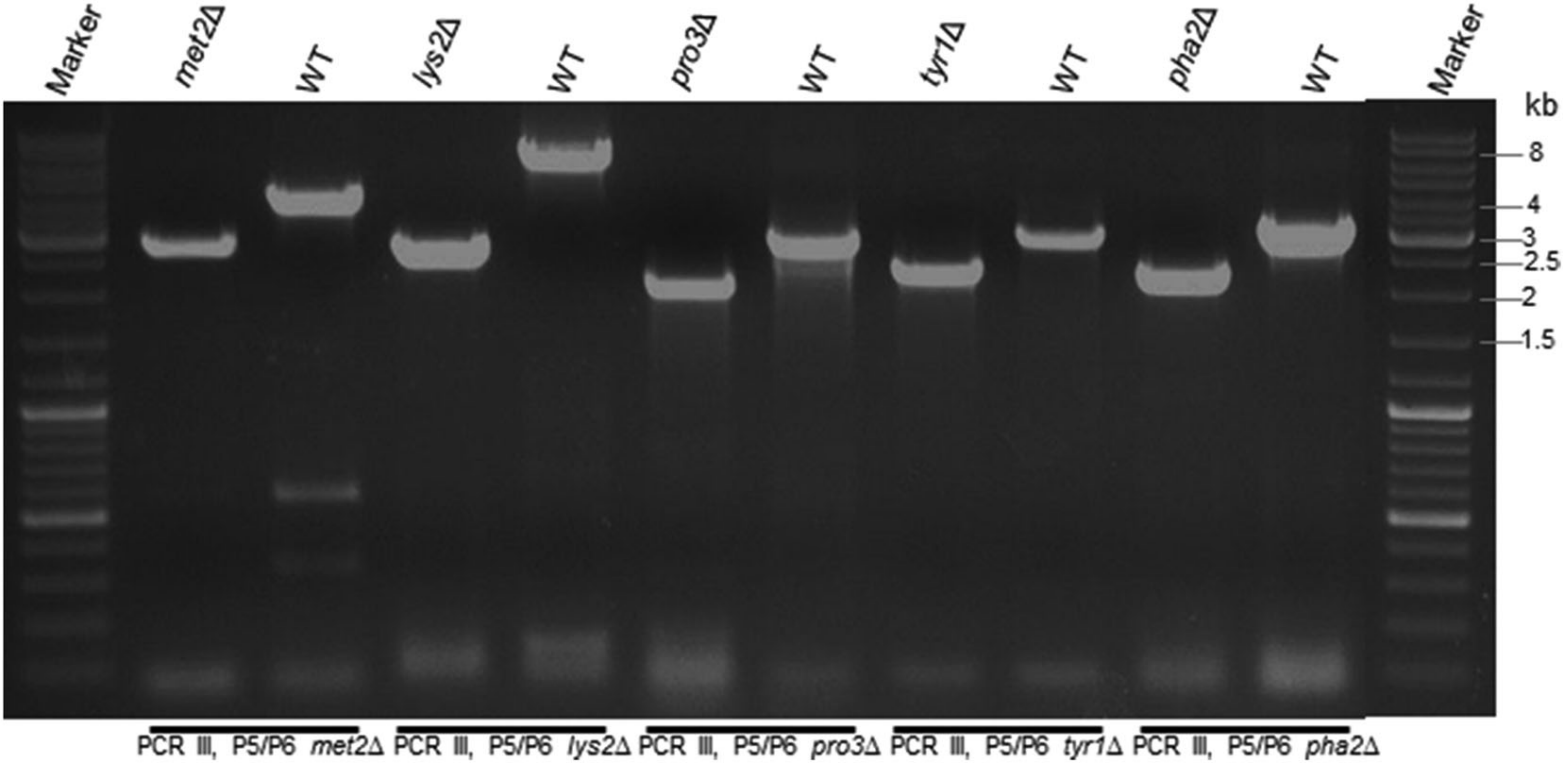
Verification of successful gene knockout by PCR analysis. The results for wild type and knockout strains are shown using knockout specific primer pairs P5/P6 (PCR III of Figure 2). Marker, GeneRuler DNA Ladder Mix (Thermo Scientific); WT, wild type CBS 7435

**Figure 4:**
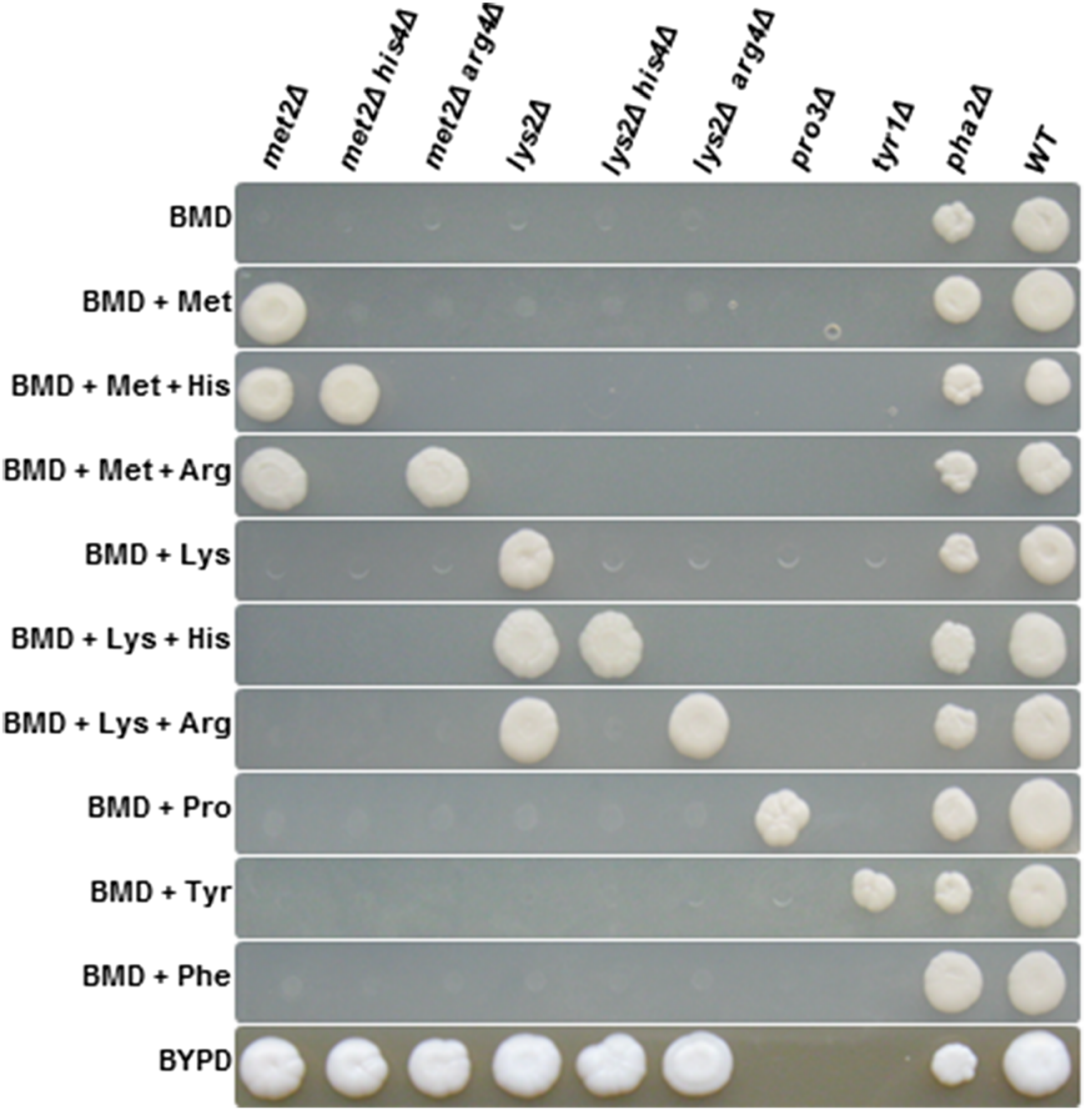
Growth behaviour of Pichia pastoris auxotrophic strains. Cells were cultivated in 96‐well deep‐well plates containing 250‐μl BMD media supplemented with the respective amino acids at 28°C, 320 rpm and 80% humidity for 24 hr. Equal number of cells (OD_600_ = 0.5) were pinned onto BMD/BYPD plates (supplemented with or without respective amino acids for selective growth), and plates were incubated for 3–4 days at 28°C

**Figure 5:**
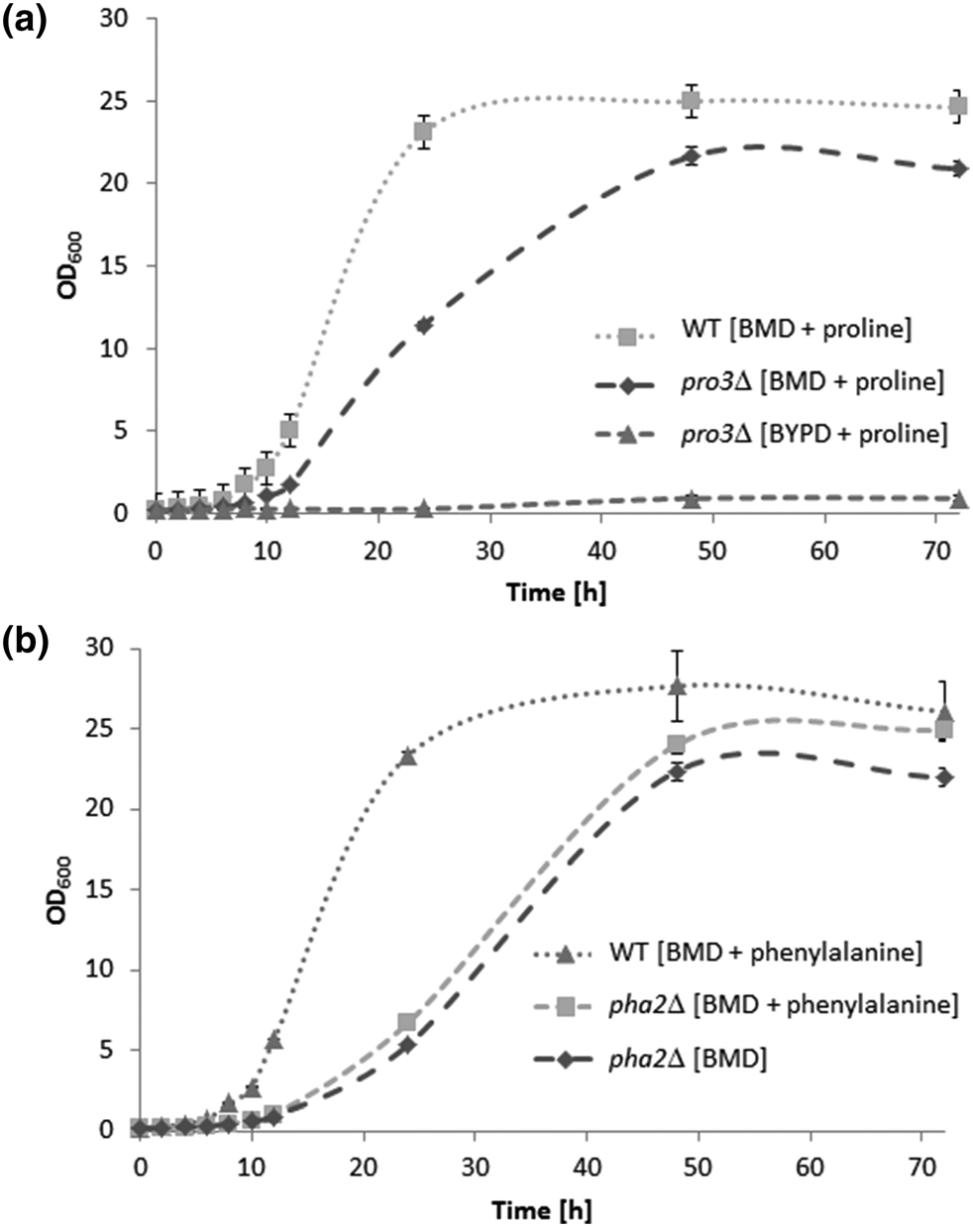
Growth rate analysis of Pichia pastoris wild type, pro3Δ, and pha2Δ strains. The strains were cultivated in 300‐ml baffled shake flasks at 28°C and 120 rpm in media as indicated, and OD_600_ was measured regularly. Experiments were performed in triplicates

Following phenotypic analysis, we confirmed that the observed amino acid auxotrophy was indeed caused by gene deletion. We isolated gDNA of the transformants to verify integration of the knockout cassette by PCR (Figure 2c). Primer pairs P5 + PAox1SeqR (PCR I) and PucSeqF+P6 (PCR II) were used to confirm the correct integration on the 5′‐ and 3′‐side, respectively. To trigger marker recycling, cells were shifted to methanol as the sole carbon source, which induced expression of Flp recombinase from P_*AOX1*_. Subsequently, Flp recombinase looped out the vector elements residing between the two FRT elements. One FRT element remained at the rearranged locus, flanked by the two *Sfi*I recognition sites. Marker recycling efficiencies for Flp‐mediated recombination were determined by testing single colonies for their resistance to Zeocin^™^ after 24 and 48 hr of induction in BMM media and were found to be 50% and ≥95%, respectively. We further verified this rearrangement by performing control PCRs with primer pairs P5 + P6 (PCR III) and P7 + P8 (PCR IV) and by sequencing the products of PCR III. Representative results of PCR III are shown in Figure 3. All constructed strains (with successfully recycled markers) and their genotypes are listed in Table 2. Growth rates were determined as described in Section 2 and prove that most knockout strains grew at wild type‐like levels. Growth phenotypes of auxotrophic strains were confirmed on selective media (Figure 4). As expected, only *pro3*Δ and *tyr1*Δ knockout strains did not grow on BYPD. All knockout strains grew on minimal media supplemented with the respective amino acids. The growth phenotypes of *met2*Δ and *lys2*Δ knockout strains have been described previously (Austin et al., 2011; Thor et al., 2005). We recorded growth curves for the *pro3*Δ knockout strain on BMD and BYPD, both supplemented with proline (Figure 5a). The *pro3*Δ knockout strain grew to high cell densities but showed a longer lag phase than the wild type strain.

**Table 2:** Strains used and constructed during this study

Pichia Pool strain collection #	Genotype	Knockout vector used	Specific growth rate (YPD media)	Reference
CBS 7435	WT	—	0.29 ± 0.00	(Näätsaari et al., 2012)
Pp3520	*his4*Δ	—	—	(Näätsaari et al., 2012)
Pp3521	*arg4*Δ	—	—	(Näätsaari et al., 2012)
Pp3445	*aox1*Δ	—	—	(Näätsaari et al., 2012)
Pp7030	*met2*Δ	pPpKC1_Met2	0.28 ± 0.00	This study
Pp7031	*arg4*Δ and *met2*Δ	pPpKC1_Met2	0.30 ± 0.00	This study
Pp7032	*his4*Δ and *met2*Δ	pPpKC1_Met2	0.30 ± 0.00	This study
Pp7033	*lys2*Δ	pPpKC1_Lys2	0.32 ± 0.01	This study
Pp7034	*arg4*Δ and *lys2*Δ	pPpKC1_Lys2	0.28 ± 0.00	This study
Pp7035	*his4*Δ and *lys2*Δ	pPpKC1_Lys2	0.27 ± 0.00	This study
Pp7036	*pro3*Δ	pPpKC1_Pro3	0.28 ± 0.01	This study
Pp7037	*tyr1*Δ	pPpKC1_Tyr1	0.27 ± 0.00	This study
Pp7029	*pha2*Δ	pPpKC1_Pha2	0.16 ± 0.00	This study
Pp6668	*sub2*Δ	pPpKC1_sub2	0.35 ± 0.01	This study
Pp6911	*his4*Δ and *pep4*Δ	pPpKC3_pep4	0.34 ± 0.00	This study
Pp6912	*prb1*Δ	pPpKC1_prb1	0.32 ± 0.00	This study
Pp6676	*prc1*Δ	pPpKC1_prc1	0.35 ± 0.00	This study
Pp6686	*yps1*Δ	pPpKC1_yps1	0.34 ± 0.01	This study
Pp6671	*yps2*Δ	pPpKC1_yps2	0.35 ± 0.00	This study
Pp6907	*yps7*Δ	pPpKC1_yps7	0.34 ± 0.00	This study
Pp6909	*kex1*Δ	pPpKC1_kex1	0.34 ± 0.00	This study
Pp6910	*his4*Δ and *kex2*Δ	pPpKC3_kex2	0.30 ± 0.00	This study
Pp6673	*prtP*Δ	pPpKC1_prtP	0.34 ± 0.00	This study
Pp6687	*ctse*Δ	pPpKC1_ctse	0.34 ± 0.01	This study
Pp6669	*kpx1*Δ	pPpKC1_kpx1	0.35 ± 0.00	This study
Pp6906	*kpx4*Δ	pPpKC1_kpx4	0.32 ± 0.02	This study
Pp6670	*kpx8*Δ	pPpKC1_kpx8	0.32 ± 0.01	This study
Pp6908	*kpx10*Δ	pPpKC1_kpx10	0.32 ± 0.00	This study
Pp6677	*kpx17*Δ	pPpKC1_kpx17	0.32 ± 0.00	This study
Pp6680	*kpx20*Δ	pPpKC1_kpx20	0.32 ± 0.00	This study
Pp6681	*kpx21*Δ	pPpKC1_kpx21	0.37 ± 0.00	This study
Pp6684	*kpx24*Δ	pPpKC1_kpx24	0.36 ± 0.00	This study
Pp6685	*kpx25*Δ	pPpKC1_kpx25	0.36 ± 0.00	This study
Pp7013	*his4*Δ, *pep4*Δ, and *prb1*Δ	pPpKC1_prb1	0.25 ± 0.00	This study
Pp7076	*yps2*Δ and *yps1*Δ	pPpKC1_yps1	0.33 ± 0.00	This study
Pp7077	*yps7*Δ and *yps2*Δ	pPpKC1_yps2	0.32 ± 0.00	This study
Pp7078	*his4*Δ, *kex2*Δ, and *yps1*Δ	pPpKC1_yps1	0.24 ± 0.01	This study
Pp7079	*his4*Δ, *pep4*Δ, and *kex2*Δ	pPpKC3_kex2	0.25 ± 0.00	This study
Pp7080	*his4*Δ, *pep4*Δ, *prb1*Δ, and *kex2*Δ	pPpKC3_kex2	0.17 ± 0.00	This study

**Figure 6:**
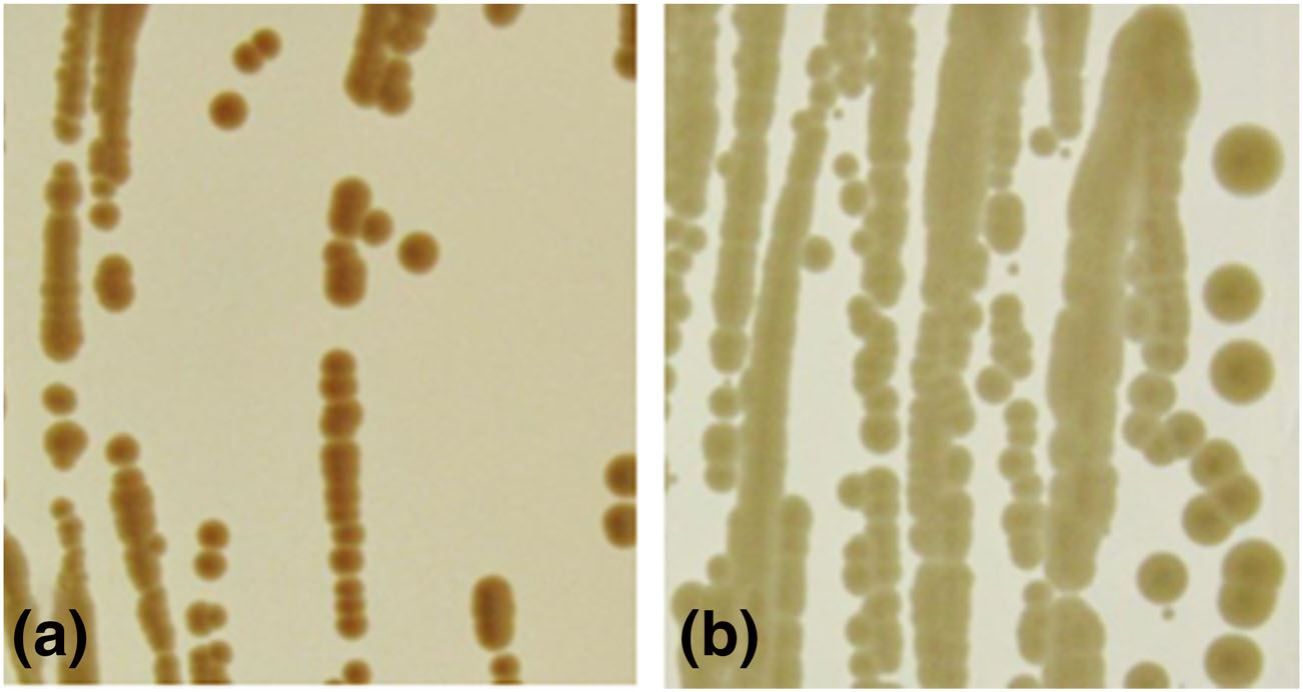
Phenotype of CBS 7435 pha2Δ (Pp7029). Growth on (a) buffered minimal media and (b) buffered minimal media supplemented with phenylalanine (150 μg/ml) after ~10 days of incubation at 28°C

We were surprised to find that the *pha2*Δ strain, which we expected to be deficient in phenylalanine biosynthesis, grew on minimal media lacking this amino acid (Figure 4). From different kingdoms of life, two pathways for the synthesis of phenylalanine are known to exist, starting either from arogenate or from phenylpyruvate. In S. cerevisiae, the only known route to phenylalanine starts from phenylpyruvate, which is produced from prephenate through the action of prephenate dehydratase (Braus, 1991). We attempted to generate strains auxotrophic for phenylalanine by deleting *PHA2*, the gene encoding prephenate dehydratase. Unexpectedly, we observed a leaky and retarded growth phenotype of the *pha2*Δ knockout strain on minimal medium (Figures 4 and 5B). Colonies turned pink after approximately 10 days on plate, which was not the case if supplemented with phenylalanine (Figure 6). These findings hint at the existence of more than one route for the biosynthesis of this aromatic amino acid in *P. pastoris*.

### 3.4 Construction and characterization of protease‐deficient strains

We selected 20 different, either already identified or putative protease genes annotated from CBS 7435 genome sequencing (Küberl et al., 2011), to be knocked out using our deletion vectors. Beside the single knockout of 20 protease genes, we also generated double and triple knockout strains, as summarized in Table 2. Targeting efficiencies are listed in Table 1 and, similar to the deletion of biosynthetic genes, vary based on the gene to be knocked out. As described for auxotrophic knockout strains, marker recycling was induced and confirmed by PCR and sequencing. Unexpectedly, we were not able to delete *PEP4* and *KEX2* using pPpKC1 (Zeocin^™^ resistance marker). Pep4 is the master vacuolar aspartyl protease and was described to activate itself and other proteases, such as proteinase B (Prb1) and carboxypeptidase Y (Prc1; Rothman, Hunter, Valls, & Stevens, 1986). Kex2 is involved in processing of signal peptides in the secretory pathway (Fuller, Brake, & Thorner, 1989; Fuller, Sterne, & Thorner, 1988), which is why deletion of these proteases may be detrimental to cell viability. In order to avoid negative effects of Zeocin^™^ in the selection process, we decided to change the marker in the knockout cassette to *HIS4*, which immediately resulted in obtaining the correct knockout strains. To investigate the effects of Zeocin^™^ on cellular fitness of *pep4*Δ and *kex2*Δ strains, serial dilution of the knockouts were plated on 5 μg/ml Zeocin^™^. Both knockout strains showed increased sensitivity to Zeocin^™^ compared with the wild type (data not shown). Knockout of *PEP4* (Pan et al., 2011) and *KEX2* (Werten & De Wolf, 2005) using Zeocin^™^ as a selection marker were reported earlier, but in these studies, expression of the marker gene was driven by P_*TEF1*_. This promoter is significantly stronger than the P_*ARG4*_ promoter we used for marker expression (Schirmaier & Philippsen, 1984). Lower expression levels of the Zeocin^™^ resistance gene may be the reason why we did not succeed in obtaining *pep4*Δ and *kex2*Δ using our vector.

**Figure 7:**
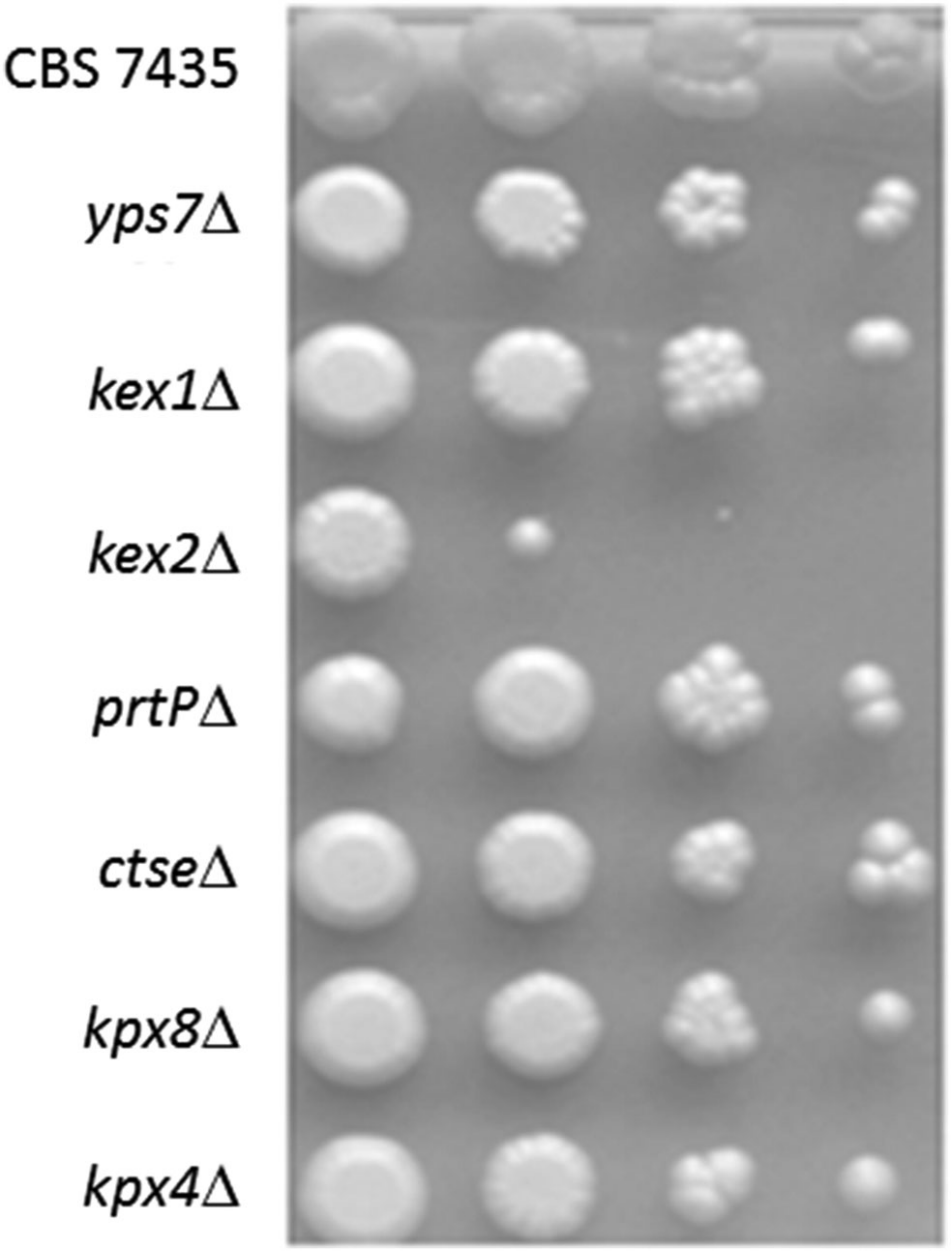
CBS 7435 his4Δ kex2Δ (Pp6910) shows growth defect on YPD plates. Knockout strains were grown to OD_600_ of 1 in YPD, diluted 10‐fold, and spotted on YPD plates. Depicted are results for knockout strains yps7Δ, kex1Δ, kex2Δ, prtPΔ, ctseΔ, kpx8Δ, and kpx4Δ. All other protease knockout strains behaved similar as the wild type

Overall fitness of protease‐deficient strains was not massively harmed if grown on liquid media (Table 2), most probably due to overlapping functions and high redundancy of the >300 proteases annotated in *P. pastoris* (Sturmberger et al., 2016). However, by comparing growth of protease‐deficient strains on YPD plates, we observed reduced growth of the *kex2*Δ knockout strain (Figure 7). Kex2 is a transmembrane calcium‐dependent protease, which cleaves on the carboxyl side of Lys‐Arg and Arg‐Arg sequences in polypeptide precursors. Mammalian Kex2 homologues, known as furin, PC1/3, PC2, PC4, PACE4, PC5/6, and PC7/LPC were shown to process hormone and neuropeptide precursors, growth factors, receptors, metallo‐ and aspartyl proteases, envelope glycoproteins of many viruses including HIV, and bacterial toxins such as anthrax‐protective antigen (Thomas, 2002). Defects in these genes have been associated with diseases such as diabetes and cancer (reviewed by (Bassi, Fu, Lopez de Cicco, & Klein‐Szanto, 2005)). In S. cerevisiae, Kex2 is mainly involved in activating proproteins of the secretory pathway, for example, the yeast pheromone α‐factor and killer toxin (Fuller et al., 1988). Studies of S. cerevisiae strains lacking *KEX2* revealed minor growth defects and higher sensitivity to low temperatures, hyperosmolarity, and diverse chemicals (Oluwatosin & Kane, 1998), indicating the essential role of this protease.

## 4 DISCUSSION

Taken together, we were able to confirm the feasibility of our knockout vector system by successfully targeting five amino acid biosynthesis and 20 known or putative protease genes. The clear advantage of our vector‐based approach for efficient construction of knockout cassettes is the possibility to amplify the construct in vivo in E. coli prior to transformation. This strategy reduces the risk of nucleotide mutations that are likely to accumulate during extensive rounds of PCR amplification. Our knockout vector system allows straightforward tailoring to the gene of interest and the *P. pastoris* strain used. The target homology regions can easily be exchanged in a single cloning step. Likewise, the selection marker of the vector can be varied as required, meaning that one could, for example, exchange *HIS4* in pPpKC3 for *LYS2* and use the new knockout cassette in a *lys2*Δ strain. Thanks to the current advances in identifying mating factor genes and the generation of heterothallic *Pichia pastoris* strains used for mating and sporulation (Heistinger et al., 2018; Heistinger, Gasser, & Mattanovich, 2017), classical yeast genetics applying tetrad analysis will soon be possible. We envision our deletion plasmids to be used to generate knockout strains that can be mated and analysed by tetrad dissection. We further suggest to use our vectors in combination with a recently developed CRISPR/Cas9 approach (Weninger et al., 2018), which still requires the cloning of repair cassettes of several kbs. Genome editing of *P. pastoris* applying CRISPR/Cas9 is working much more efficiently in a *ku70*Δ strain background than in wild type cells, which is why the use of efficiently working selection markers in the repair cassettes drastically facilitates identification of strains with correct genetic alterations. With minor modifications, the vector system presented here could also be exploited for targeted integration of protein expression cassettes at any defined locus. Moreover, the possibility to recycle the selection marker allows a cascade of gene deletions or expression cassettes to be integrated into the genome, even after tetrad analysis or CRISPR/Cas9 mediated engineering. This quality makes our vector system not only a convenient tool in fundamental research but especially complex metabolic engineering projects.

## CONFLICT OF INTERESTS

The authors declare no conflict of interest.

## Supporting information

Table S1: Primers used in this study

Table S2: Elements of E. coli/P. pastoris gene knockout shuttle vectors and their function.

## ENDNOTE

1 To obtain plasmids and strains from the *Pichia Pool*, please contact Prof. Helmut Schwab: helmut.schwab@tugraz.at.
