# Supplementary material for: Pichia pastoris protease‐deficient and auxotrophic strains generated by a novel, user‐friendly vector toolbox for gene deletion
Source: Yeast. 2019 Jul 30;36(9):557–70. doi: 10.1002/yea.3426 (PMC6771850; doi:10.1002/yea.3426)
Supplement: Supplementary file 2 — Table S2: Elements of E. coli/P. pastoris gene knockout shuttle vectors and their function. [file YEA-36-557-s002.docx]

**Table S2: Elements of *E. coli*/*P. pastoris* gene knockout shuttle vectors and their function.**

| **Elements** | **Origin** | **Function** |
| --- | --- | --- |
| P_Aox1 | *Pichia pastoris* CBS7435 | *P. pastoris* *AOX1* promoter for expression of Flippase |
| Flippase^a^ | *Saccharomyces cerevisiae* BY4741 | Site-specific FLP recombinase, recycling of the marker genes (mutated to remove certain restriction sites) |
| Aox1_TT | *Pichia pastoris* CBS7435 | Transcription terminator of *AOX1* gene in *P. pastoris* for Flippase transcription termination |
| P_Arg4 | *Pichia pastoris* CBS7435 | *ARG4* promoter for expression of selection marker genes in *P. pastoris* |
| Arg4_TT | *Pichia pastoris* CBS7435 | *ARG4* transcription terminator for expression of selection marker genes |
| EM 72 Syn B | Synthetic sequence, amplified from pPpT4 [10] | Constitutive prokaryotic promoter; drives expression of antibiotic resistance genes in *E. coli* |
| *Sh_ble* | Synthetic gene, amplified from pPpT4 [10] | Confers resistance to antibiotic Zeocin |
| *Arg4*_CDS | *Pichia pastoris* CBS7435 | *P. pastoris* wild type gene coding for argininosuccinate lyase; selection marker |
| KanMX | *KanMX* amplified from pPpT4_Kan [10] | *KanMX* gene; selection marker |
| *His4*_CDS | *Pichia pastoris* CBS7435 | *P. pastoris* *HIS4* wild type gene; selection marker |
| pUC Ori | pUC8 (Genbank Acc. Nr. L08959) | pUC origin of replication for plasmid maintenance in *E. coli* |
| FRT | Synthetic FRT site | FLP recombinase recognition sequence for marker recycling |
| *bla*_cds | *β-lactamase* gene from pUC8 (Genbank Acc. Nr. L08959) | Ampicillin resistance in *E. coli;* selection marker |
| Stuffer | Synthetic gene HRP0 (Genbank Acc. Nr. HE963800.1) | For easier confirmation of restriction with *SfiI* restriction enzyme |

1. Three restriction sites, namely *SwaI*, *EcoRI* and *NdeI* were mutated by creating silent mutations with overlap extension PCR.
